# Maternal Exposure to Occupational Solvents and Childhood Leukemia

**DOI:** 10.1289/ehp.7707

**Published:** 2005-03-03

**Authors:** Claire Infante-Rivard, Jack Siemiatycki, Ramzan Lakhani, Louise Nadon

**Affiliations:** ^1^Department of Epidemiology, Biostatistics, and Occupational Health, Faculty of Medicine, McGill University, Montréal, Québec, Canada;; ^2^Département de Médecine sociale et préventive, Université de Montréal, Montréal, Québec, Canada;; ^3^Institut National de la Recherche Scientifique–Institut Armand-Frappier, Laval, Québec, Canada

**Keywords:** acute lymphoblastic leukemia, child, childhood leukemia, maternal occupational exposure, solvents

## Abstract

Many organic solvents are considered probable carcinogens. We carried out a population-based case–control study including 790 incident cases of childhood acute lymphoblastic leukemia and as many healthy controls, matched on age and sex. Maternal occupational exposure to solvents before and during pregnancy was estimated using the expert method, which involves chemists coding each individual’s job for specific contaminants. Home exposure to solvents was also evaluated. The frequency of exposure to specific agents or mixtures was generally low. Results were generally similar for the period ranging from 2 years before pregnancy up to birth and for the pregnancy period alone. For the former period, the odds ratio (OR), adjusted for maternal age and sex, for any exposure to all solvents together was 1.11 [95% confidence interval (CI), 0.88–1.40]. Increased risks were observed for specific exposures, such as to 1,1,1-trichloroethane (OR = 7.55; 95% CI, 0.92–61.97), toluene (OR = 1.88; 95% CI, 1.01–3.47), and mineral spirits (OR = 1.82; 95% CI, 1.05–3.14). There were stronger indications of moderately increased risks associated with exposure to alkanes (C5–C17; OR = 1.78; 95% CI, 1.11–2.86) and mononuclear aromatic hydrocarbons (OR = 1.64; 95% CI, 1.12–2.41). Risk did not increase with increasing exposure, except for alkanes, where a significant trend (*p* = 0.04) was observed. Home exposure was not associated with increased risk. Using an elaborate exposure coding method, this study shows that maternal exposure to solvents in the workplace does not seem to play a major role in childhood leukemia.

Acute lymphoblastic leukemia (ALL) is the most frequent form of cancer in children (National Cancer Institute of Canada 2004). At this time, there is only limited knowledge and evidence on environmental or other risk factors contributing to the incidence of ALL. Some convincing data show that ALL can arise *in utero* because characteristic chromosome translocations that generate chimeric fusion genes unique for each patient’s leukemic clone are found at birth (Greaves 2002); therefore, the pregnancy and periconceptional periods are exposure windows of primary interest to study risk factors that could be involved in childhood ALL.

Fetal exposure to chemical agents is likely to come primarily from maternal exposure at work. Among chemical agents with carcinogenic potential and to which a substantial proportion of workers are likely to be exposed are organic solvents. In a multisite case–control study of cancer patients in Montréal, Canada, an estimated 40% of men had been occupationally exposed to at least one solvent over the course of their work careers (Siemiatycki 1991). A solvent is any substance capable of dissolving another substance to form a uniformly dispersed mixture or solution (Stacey 1993). In industrial processes, water, a polar solvent, is often incapable of dissolving a large number of substances, and therefore organic liquids are used. The expressions “industrial solvents” and “organic solvents” are conventionally applied to these organic liquids (Stacey 1993).

Previous studies assessing parental occupational exposures for ALL have not always evaluated maternal exposures or have done so in studies of small size where a substantial proportion of mothers were homemakers, often leading to few usable results (Colt and Blair 1998). More recent and larger studies have included an assessment of maternal exposures; one reported “solvents” as an exposure category (Schuz et al. 2000), whereas another reported results on specific solvents or groups of solvents; but in both studies, exposure assignment seems to have been directly based on parents’ self-reporting (Shu et al. 1999). Recently, McKinney at al. (2003) used parental self-report and a group of professionals to assign exposure to “occupational groups” (a mixture of occupations, industries, and groups of agents). Overall, results from previous studies that reported on solvents and leukemia have not been consistent. We conducted a case–control study of childhood leukemia using the expertise of trained chemists to determine maternal exposure to occupational solvents.

## Materials and Methods

### Case ascertainment.

Details of the study have been described elsewhere (Infante-Rivard 2003; Infante-Rivard et al. 2000, 2001). Briefly, cases of ALL diagnosed between 1980 and 2000 in the province of Québec, Canada, were recruited from tertiary care centers designated by governmental policy to hospitalize and treat children with cancer in the province. Tracing cases from these hospitals is equivalent to population-based ascertainment. Between 1980 and 1993 cases 0–9 years of age at diagnosis were recruited for study; between 1994 and 2000 case selection included those up to 14 years of age at diagnosis. A case was determined to have ALL (*International Classification of Diseases*, *9th Revision*, code 204.0) (World Health Organization 1975) on the basis of a clinical diagnosis by an oncologist or a hematologist. Because cancer care is covered under a universal health plan, we believe that a negligible number of children, if any, were treated outside the province.

### Control selection.

Population-based controls (one per case) were matched on sex and age at the time of diagnosis (calendar date) and thus were concurrently selected. From 1980 to 1993, the population-based controls were chosen from family allowance files (Régie des Rentes du Québec, Québec, Canada). The family allowance is a government stipend awarded to all families with children living legally in Canada. This source of data was the most complete census of children for the study years. According to the expected distribution of cases based on matching criteria, a list of 10 potential controls was randomly chosen from the lists. Between 1994 and 2000, we used the provincial universal health insurance files (Régie de l’Assurance Maladie du Québec, Québec, Canada) as a source for controls, which is an equivalently complete census of children. It was a better source of data for that time period because family allowances were more often directly deposited in the mother’s bank account, which meant that the home address was no longer available in the file. We proceeded the same way to obtain potential controls.

### Study participants.

Children who were adopted, who lived in foster families, whose families spoke neither French nor English, who did not reside in Canada, or whose parents were both unavailable for interview were excluded. We identified 848 eligible cases, and interviewed the parents of 790 (93.1%); of 916 eligible controls, 790 parents were interviewed (86.2%). The reasons for nonparticipation were confidential telephone number, refusal to participate, or inability to trace the family. The study was approved by each hospital’s institutional review board as well as by the provincial agency regulating access to public databases with nominal information. We requested that the parents return a signed informed consent form for the interview.

### Data collection.

Soon after the anticipated reception of a letter introducing participants to the general purpose of the study, trained interviewers contacted the parents to schedule an appointment for the interview, which was administered by telephone using structured questionnaires. Questionnaires were reviewed as they came in, and feedback was given regularly to interviewers. One questionnaire addressed general risk factors and potential confounders. To assess maternal occupational risk factors, the procedure was as follows: A complete job history was obtained from the mother for the period ranging from 18 years of age to the end of pregnancy. This information included the job title and dates on this job, and the type of industry and its name and address. For each job held by the mother from 2 years before pregnancy and up to birth of the index child, a semistructured questionnaire was used to probe the details of each job; as previously described (Goldberg et al. 2001), the information collected included the company’s activities; raw materials used; machines used; goods produced; responsibilities for machine maintenance; type of room or building in which the woman worked; activities of workmates; presence of gases, fumes, dusts, biocides, oils, solvents, and ionizing and nonionizing radiation sources; use of area or personnel protective equipment; and a detailed open-ended description of the woman’s typical activities at work. In addition, for frequent job titles and/or jobs with a significant potential for occupational exposures (e.g., nurse, sewing machine operator, hairdresser, waitress, cook, textile dry cleaner, knitting and weaving operator), a specialized questionnaire was also administered that probed more deeply into the specific tasks, the time spent at them, specific exposures related to these tasks, and the environment in which they were conducted.

### Exposure coding.

Exposure coding was carried out by a team of chemists and industrial hygienists who have many years of experience in exposure assessment in community-based case–control studies. They first assigned each occupation to standard Canadian industrial titles (at the three-digit level) and job titles (at the seven-digit level) (Statistics Canada 1980, 1992). The next step was to determine whether there was or was not exposure to specific solvents or chemical mixtures with solvents (listed in Table 1; discussed further below); the complete list of chemicals that were coded includes > 300 items, but the focus here is on solvents because these were the chemicals of primary interest of the study. The strategy to code exposures from individual job histories is termed the “expert method” in the occupational epidemiology literature (Teschke et al. 2002) and has been described previously (Gérin et al. 1985; Siemiatycki et al. 1987). Briefly, experienced chemists use all the available information provided by the study subject, information accumulated from coding exposures for thousands of jobs held in the same geographical area (albeit for men) (Siemiatycki 1991), and their personal knowledge or consultants’ knowledge of the industries. Chemists were blind to the case/control status.

### Home exposure to solvents.

The general questionnaire included items to assess exposure to solvents in the home: hobbies, such as model building, furniture stripping, and types of art work; activities carried out in and around the home with the potential for similar exposure, such as electronic and motor vehicle repair; and painting in the home. For each question, we asked who carried out the activity and during what time period, specified as 1 year before pregnancy, during pregnancy, and from birth to the reference date.

### Statistical analysis.

Two time periods were defined: from 2 years before pregnancy up to birth, and during the specific pregnancy period. We used conditional logistic regression to estimate odds ratios (ORs) and 95% confidence intervals (CIs). Each agent, mixture, and family was analyzed in a separate model, and the analyses were adjusted for maternal age and level of schooling. Results are first presented contrasting any exposure with no exposure. For the exposure period ranging from 2 years before pregnancy up to birth, we repeated the analysis contrasting “any exposure” with “no exposure” but this time taking into account the chemist’s confidence factor in coding. In the latter analysis, if the chemist had coded the exposure as “possible” (vs. “probable” and “definite”), exposure was assigned to the “no exposure” category. For the same time window, we also conducted an analysis with three levels of exposure: level 0 (baseline), no exposure (defined as none coded or exposure coded with a “possible” confidence); level 1, some exposure (exposure resulting in concentration × frequency < 4), and level 2, greater exposure (concentration × frequency ≥4). Finally, we used a model that includes all specific agents and mixtures. We analyzed residential exposure to solvents in the household as never/ever for each question.

## Results

The distribution of sociodemographic characteristics between cases and controls was quite similar (Table 2). More than 99% of mothers in both the case and control groups answered their own questionnaires. The proportion of women with gainful employment in both groups was almost equal (Table 3). Control mothers had an average of 1.33 jobs over the study period, whereas case mothers had an average of 1.29 jobs. Thirteen job titles among the 15 most frequently held jobs were similar between case and control mothers. There were more sewing machine operators and cosmetologists among case mothers than among control mothers.

## Discussion

The distributions of jobs between case and control mothers were remarkably similar, with a few exceptions, the main one being that there were more sewing machine operators in the case group. This was previously reported for the study subjects included between 1980 and 1993 (Infante-Rivard and Deadman 2003). Despite the fact that prevalence of any exposure to unspecific solvents as a whole was substantial, that to specific agents or mixtures was low and even lower at the highest levels for these agents. For the “solvents” category, cases and controls had a similar exposure prevalences. Among the specific agents and mixtures to which mothers were exposed before or during pregnancy and that were associated with increased risk of ALL were 1,1,1-trichloroethane, toluene, mineral spirits, leaded gasoline, and possibly methylene chloride and methyl ethyl ketone. Among the chemical families, there were stronger indications of increased risk for alkanes (C5–C17) and mononuclear aromatic hydrocarbons.

In a recent review on organic solvents and cancer, Lynge et al. (1997) reported that there is some evidence for an increased risk of cancer with toluene, 1,1,1-trichloroethane, and methylene chloride, although none is classified yet as a carcinogen by any regulatory agency. The U.S. National Institute for Occupational Safety and Health (NIOSH 1978) issued a bulletin on chloroethanes stating that 1,1,1-trichloroethane should be treated in the work-place with caution because of its structural similarity to other chloroethanes shown to be carcinogenic in animals. Mineral spirits are refined petroleum solvents that include varnish makers’ and painters’ naphtha, Stoddard solvent, and white spirits (Siemiatycki 1991). They are largely composed of saturated hydrocarbons (or alkanes, which in liquid form are C5–C17), but also include a small proportion of benzene. Although benzene is a recognized leukemogenic agent [International Agency for Research on Cancer (IARC) 1987], there is little information on the carcinogenicity of mineral spirits as such; a Swedish study reported an increased risk of acute leukemia among painters (Lindquist et al. 1987), but painters may be exposed to a greater extent to other solvents such as toluene and xylene. Leaded gasoline, another mixture that showed indications of increasing risk, is a mixture of hydrocarbons used as fuel for automobiles; it also contains benzene and toluene. A fairly consistent link between fathers with occupations in motor-vehicle–related occupations and childhood leukemia has been reported (Colt and Blair 1998); however, such occupations involve exposure to a variety of chemicals, including polycyclic aromatic hydrocarbons such as benzo[*a*]pyrene, which is considered a probable or suspected carcinogenic agent by regulating agencies (IARC 1983).

Results from studies on maternal occupational exposures and childhood ALL published between 1980 and 1997 (Feingold et al. 1992; Gold et al. 1982; Hemminki et al. 1981; Lowengart et al. 1987; Magnani et al. 1990; McKinney et al. 1987; Olsen et al. 1991; Shu et al. 1988; Van Steensel-Moll et al. 1985) have been reviewed before (Colt and Blair 1998). In all these studies except that by Feingold et al. (1992), exposure was defined as having an occupation or belonging to an exposed industry, or occasionally, exposure to a specific agent was reported, as determined by maternal reporting. This strategy was also used in a more recent study from Germany (Schuz et al. 2000) not included in the review. On the other hand, Feingold et al. (1992) used a general job-exposure matrix to determine exposure to a list of specific agents; unfortunately, this study was very small (~ 60 cases of ALL and 60 controls), and results were inconclusive. In a more recent and large study from the United States (Shu et al. 1999), mothers reported specific exposures as well as the approximate length of time spent being exposed to a particular agent. No additional strategy to code exposure involving chemists or similar experts is explicitly described, so it is assumed that the self-reported exposures were used as such. Finally, in another recently published and large study, this time from the United Kingdom (McKinney et al. 2003), occupations and industries were coded according to standard classifications; in addition, a panel of experts, including a hygienist, created 31 occupational groups that were said to be homogeneous for specific exposures. The occupational groups used in the analysis include job titles (e.g., leather workers), sectors of activity (e.g., agriculture), and agents such as solvents and hydrocarbons (skin/epidermal or inhaled particulate). The method to create the groups is not detailed in the report. Because of the different ways to classify exposures and the multiple classifications used (even for job titles or industries, each study using their respective national classification), results are difficult to compare. However, in the three more recent studies (McKinney et al. 2003; Schuz et al. 2000; Shu et al. 1999), an explicit “solvents” category was used, and results are as follows: Schuz et al. (2000) report an OR of 1.2 (95% CI , 0.9–1.7) for exposure to solvents during periconception and a similar result during pregnancy. Shu et al. (1999) report an OR of 1.8 (95% CI, 1.3–2.5) for exposure to “possible organic solvents” during preconception (2 years before conception) and a similar result during pregnancy. McKinney et al. (2003) report an OR associated with exposure to solvents at periconception of 1.0 (95% CI, 0.66–1.51); however, exposure to “dermal hydrocarbons” had an OR of 2.16 (95% CI, 1.16–4.02).

The present study uses the most detailed and elaborate exposure assessment method in comparison with previous studies. The expert method used here has been found to have good validity (Fritschi et al. 2003). Its steps have been clearly detailed (Siemiatycki 1991), and results using this method to uncover carcinogens in community-based case–control studies have been abundantly published (Aronson et al. 1996; Parent et al. 2000). In this study, all specific agents associated with increased risk have also been associated with increased risks of cancer in previous studies. Although results for some specific agents or families indicated increased risk, this was not as clear for the general category “solvents.” The families of alkanes (C5–C17) and mono-nuclear aromatic hydrocarbons are ones that many previous studies have tried to capture by using the hydrocarbon-related occupations (Lowengart et al. 1987; McKinney et al. 2003; Olsen et al. 1991; Shu et al. 1988, 1999; Van Steensel-Moll et al. 1985). The result by McKinney et al. (2003) showing a substantial increase in risk associated with periconception dermal exposure to hydrocarbons is consistent with our own. Overall, our results on alkanes and mononuclear aromatic hydrocarbons are consistent with and reinforce previous results. However, except for alkanes, it was disconcerting to find no indication of an exposure–response relationship, an observation also reported by Shu et al. (1999).

A study reported results on home exposure to solvents and childhood ALL (Freedman et al. 2001). Only artwork at a frequency of more than four times a month was associated with an increased risk of ALL. We did not measure frequency of exposure for home solvents, but none of the ORs in our study indicated any increase in risk for the ever-exposed category.

In comparison with previous studies on parental occupational exposures and childhood ALL, this is the third largest study in terms of number of cases. Nevertheless, power is still an issue. With respect to potential biases often affecting case–control studies, in this study selection bias was unlikely: participation rates for cases and especially for controls were markedly higher (by ~ 20%) than in any of the other large studies cited. However, although the exposure assignment method used in this study seems more refined than in previous studies, it is safe to say that nondifferential misclassification of exposures affected the results and reduced our ability to uncover significant findings.

In conclusion, this study used an exposure assignment method that is among the best available for community-based case–control studies of cancer. The results gave more specific indications than previous studies and point to an increase in ALL risk associated with maternal exposure to occupational alkanes and mononuclear aromatic hydrocarbons. Nevertheless, the results are still somewhat uncertain. From a public health point of view, it was reassuring to observe that, as in all previous studies, maternal exposures were most often rare and occurred at low levels; this, of course, makes the task of uncovering effects more difficult. However, this fact should not deter us from a continued search in this direction because prenatal exposure to carcinogens as risk factors for childhood leukemia makes biologic sense, and even low levels during this period could be damaging. We are thus challenged to develop more sensitive methods to ascertain parental occupational exposures. Adding a genetic susceptibility perspective could also enhance our ability to uncover the susceptible dyads (mother and child).

## Figures and Tables

**Table 1:** Matrix of specific chemicals, complex mixtures of chemicals, and chemical families used in the analysis.

		Chemical families*^b^*						
	Code*^a^*	1	2	3	4	5	6	7
Specific chemicals								
Methanol	232		XX*^c^*					
Ethanol	233		XX					
Isopropanol	234		XX					
Ethylene glycol	235		XX					
Carbon tetrachloride	237			XX				
Chloroform	238			XX				
Methylene chloride	239			XX				
1,1,1-Trichloroethane	240			XX				
Trichloroethylene	242				XX			
Perchloroethylene	243				XX			
Ethylene dichloride	300				XX			
Acetone	248					XX		
Methyl ethyl ketone	304					XX		
Benzene	252						XX	
Toluene	253						XX	
Xylene	254						XX	
Ethyl acetate	302							XX
Diethyl ether	250							
Turpentine	280							
Carbon disulfide	266							
Butyl cellosolve	306							
Mixtures								
Mineral spirits post-1970*^d^*	202	X*^e^*					X	
Mineral spirits pre-1970*^d^*	203	X					X	
Leaded gasoline	191	X					X	
Unleaded gasoline	299	X					X	
Aviation gasoline	190	X					X	
Kerosene	195	X					X	

a These codes were used by Siemiatycki (1991) to catalogue and define the various substances, and they can thus be used to easily find additional information on these chemicals in that reference.

b Chemical families: 1, alkanes (C5–C17); 2, aliphatic alcohols; 3, chlorinated alkanes; 4, chlorinated alkenes; 5, aliphatic ketones; 6, mononuclear aromatic hydrocarbons; 7, aliphatic esters.

c XX signifies that the agent listed to the left is a member of the chemical family indicated at the top.

d Before 1970, mineral spirits contained relatively higher amounts of benzene, toluene, and xylene due to ignorance of their toxic effects.

e X signifies that the agent listed to the left contains components that are members of the chemical family indicated at the top.

**Table 2:** Demographic characteristics [no. (%)] of ALL cases and controls.

Cases	Cases (*n* = 790)	Population controls (*n* = 790)
Mother’s education		
None or primary school	34 (4.3)	25 (3.2)
Secondary school	437 (55.3)	436 (55.2)
College or university	319 (40.4)	328 (41.6)
Mother’s age at child’s birth		
< 35	721 (91.3)	743 (94.0)
≥35	69 (8.7)	47 (6.0)
Family income at diagnosis (Can$)		
≥40,000	312 (39.9)	309 (40.2)
10,000–39,000	427 (54.7)	422 (54.9)
< 10,000	42 (5.4)	38 (4.9)

**Table 3:** Distribution of job titles among mothers of ALL cases and population controls during the period ranging from 2 years before pregnancy up to birth of the index child.

	Cases	Controls
No. not working (%)	178 (22.5)	173 (21.9)
No. working	612	617
No. of jobs held (average per person)	792 (1.29)	820 (1.33)
Job titles by order of frequency (highest to lowest)		
	Secretary	Secretary
	Clerk (general office)	Clerk (general office)
	Sewing machine operator	Waitress
	Waitress	Nurse (general duty)
	Cashier (clerical)	Cashier (clerical)
	Nurse (general duty)	Teller
	Cosmetologist	Sales clerk
	Sales clerk	Elementary school teacher
	Teller	Sewing machine operator
	Elementary school teacher	Cashier (customer service)
	Baby sitter	Cosmetologist
	Receptionist	Receptionist
	Computer operator	Baby sitter
	Accountant clerk	Accountant clerk
	Nurse’s aide	Counterwoman (cafeteria)

**Table 4:** Adjusted*^a^* OR (95% CI) and ratio of discordant pairs (RDP) for maternal exposure to solvents.

	2 years before pregnancy up to birth		During pregnancy	
	OR*^b^* (95% CI)	RDP	OR (95% CI)	RDP
Specific chemicals				
Methanol	0.77 (0.41–1.47)	17:22	0.78 (0.39–1.55)	15:19
Ethanol	1.22 (0.66–2.25)	23:19	1.06 (0.55–2.03)	19:18
Isopropanol	0.96 (0.71–1.29)	85:89	0.95 (0.69–1.31)	73:78
Chloroform	0.25 (0.05–1.17)	2:8	0.25 (0.05–1.17)	2:8
Methylene chloride	1.34 (0.54–3.34)	11:8	1.25 (0.46–3.35)	9:7
1,1,1-Trichloroethane	7.55 (0.92–61.97)	7:1	4.07 (0.45–36.7)	4:1
Perchloroethylene	0.96 (0.41–2.25)	11:11	0.84 (0.30–2.34)	7:8
Acetone	1.05 (0.53–2.08)	17:16	1.13 (0.52–2.44)	14:12
Methyl ethyl ketone	—	4:0	—	4:0
Benzene	0.82 (0.22–3.06)	4:5	1.39 (0.31–6.25)	4:3
Toluene	1.88 (1.01–3.47)	29:16	2.25 (1.02–4.95)	20:9
Diethyl ether	0.50 (0.17–1.48)	5:15	0.63 (0.20–1.93)	5:8
Turpentine	1.76 (0.42–7.42)	5:3	1.76 (0.42–7.42)	5:3
Mixtures				
Mineral spirits, post-1970	1.82 (1.05–3.14)	37:20	1.66 (0.86–3.22)	24:14
Minerals spirits, pre-1970	—	5:0	—	4:0
Leaded gasoline	5.09 (0.59–43.65)	5:1	4.14 (0.46–37.16)	4:1
Unleaded gasoline	0.90 (0.30–2.71)	6:7	0.83 (0.22–3.10)	4:5
Chemical families*^c^*				
Alkanes (C5–C17)	1.78 (1.11–2.86)	48:27	1.72 (0.98–3.03)	33:19
Aliphatic alcohols	0.90 (0.68–1.18)	97:108	0.89 (0.66–1.20)	84:95
Chlorinated alkanes	1.33 (0.68–2.61)	20:15	1.05 (0.50–2.19)	15:14
Chlorinated alkenes	0.97 (0.43–2.17)	12:12	0.86 (0.33–2.25)	8:9
Aliphatic ketones	1.30 (0.68–2.50)	21:16	1.46 (0.70–3.03)	18:12
MAH	1.64 (1.12–2.41)	70:43	1.68 (1.06–2.67)	49:29
Solvents*^d^*	1.09 (0.87–1.38)	154:141	1.00 (0.78–1.28)	125:125

MAH, mononuclear aromatic hydrocarbons.

a Adjusted for maternal age and level of schooling; specific chemicals or mixtures with fewer than four exposed mothers are not shown.

b Odds ratio (95% CI) for any exposure; baseline is no exposure.

c Chemical families regroup specific chemicals that belong to a family and mixtures that have components belonging to it.

d Includes all specific chemicals and mixtures in the table.

**Table 5:** Adjusted ORs*^a^* (95% CIs), versus possible/no exposure, for levels of maternal exposure to solvents during the 2 years before pregnancy up to birth.

	Probable/definite	Level 1*^b^*	Level 2*^c^*
Specific chemicals			
Methanol	0.81 (0.43–1.55)	0.81 (0.38–1.70)	0.82 (0.25–2.77)
Ethanol	1.11 (0.59–2.08)	1.44 (0.61–3.39)	0.81 (0.32–2.07)
Isopropanol	0.97 (0.72–1.31)	0.92 (0.65–1.32)	1.09 (0.65–1.84)
Chloroform	0.16 (0.02–1.36)	0.30 (0.03–2.90)	—
Methylene chloride	3.22 (0.88–11.73)	4.68 (0.55–40.20)	2.49 (0.48–12.81)
Perchloroethylene	0.87 (0.35–2.18)	0.95 (0.35–2.55)	0.55 (0.05–6.34)
Acetone	1.11 (0.54–2.29)	0.95 (0.39–2.28)	1.55 (0.43–5.51)
Benzene	0.77 (0.17–3.48)	—	1.47 (0.25–8.85)
Toluene	1.98 (1.06–3.72)	3.19 (1.43–7.12)	0.68 (0.18–22.05)
Diethyl ether	0.63 (0.20–1.94)	0.67 (0.19–2.41)	0.51 (0.04–5.59)
Turpentine	1.76 (0.42–7.42)	1.64 (0.27–9.92)	2.00 (0.18–22.05)
Mixtures			
Mineral spirits, post-1970	1.74 (0.99–3.06)	1.60 (0.86–2.98)	2.50 (0.66–9.46)
Chemical families*^d^*			
Alkanes (C5–C17)*	1.78 (1.09–2.91)	1.56 (0.91–2.67)	3.39 (0.94–12.21)
Aliphatic alcohols	0.91 (0.69–1.20)	0.89 (0.64–1.23)	0.95 (0.60–1.51)
Chlorinated alkanes	2.00 (0.90–4.47)	2.18 (0.67–7.10)	1.86 (0.62–5.57)
Chlorinated alkenes	0.89 (0.37–2.11)	1.07 (0.41–2.80)	0.35 (0.03–3.53)
Aliphatic ketones	1.40 (0.71–2.77)	1.24 (0.54–2.84)	1.80 (0.52–6.17)
MAH	1.67 (1.13–2.48)	1.82 (1.15–2.87)	1.32 (0.62–2.80)
Solvents*^e^*	1.11 (0.88–1.40)	1.11 (0.85–1.46)	1.11 (0.75–1.63)

MAH, mononuclear aromatic hydrocarbons.

a Adjusted for maternal age and level of schooling.

b Defined as concentration × frequency < 4; baseline is possible or no exposure.

c Defined as concentration × frequency ≥4.

d Chemical families regroup specific chemicals that belong to a family and mixtures that have components belonging to it.

e Includes all specific chemicals and mixtures in the table.

* *p*-Value for trend = 0.04.
